# Design and Performance of an InAs Quantum Dot Scintillator with Integrated Photodetector

**DOI:** 10.3390/s24227178

**Published:** 2024-11-08

**Authors:** Tushar Mahajan, Allan Minns, Vadim Tokranov, Michael Yakimov, Michael Hedges, Pavel Murat, Serge Oktyabrsky

**Affiliations:** 1 College of Nanotechnology, Science and Engineering, University at Albany, Albany, NY 12203, USA; tmahajan@albany.edu (T.M.); aminns@albany.edu (A.M.); vtokranov@albany.edu (V.T.); myakimov@albany.edu (M.Y.); 2 Fermi National Accelerator Laboratory, Batavia, IL 60510, USA; mhedges@fnal.gov (M.H.); murat@fnal.gov (P.M.)

**Keywords:** novel detector technology, scintillator detector, thin detector, integrated photodetector, semiconductor radiation detectors

## Abstract

A new scintillation material composed of InAs quantum dots (QDs) hosted within a GaAs matrix was developed, and its performance with different types of radiation is evaluated. A methodology for designing an integrated photodetector (PD) with a low defect density and that is optically matched to the QD’s emission spectrum is introduced, utilizing an engineered epitaxial InAlGaAs metamorphic buffer layer. The photoluminescence (PL) collection efficiency of the integrated PD is examined using two-dimensional scanning laser excitation. The detector response to 5.5 MeV α-particles and 122 keV photons is presented. Yields of 13 electrons/keV for α-particles and 30–60 electrons/keV for photons were observed. The energy resolution of 12% observed with α-particles was mainly limited by noise- and geometry-related optical losses. The radiation hardness of an InAs QDs hosted within GaAs and a wider band gap AlGaAs ternary alloy was studied under a 1 MeV proton implantation up to a 10^14^ cm^−2^ dose. The integrated PL responses were compared to evaluate PL quenching due to non-radiative defects. The QDs embedded in the AlGaAs demonstrated improved radiation hardness compared to QDs in the GaAs matrix and in the InGaAs quantum wells.

## 1. Introduction

Ultrafast high light yield scintillation detectors are critically needed for multiple high-energy physics applications, as well as for medical imaging and security purposes [1,2,3]. Semiconductor materials, in particular doped group II–VI scintillators, are known for their exceptional light yield and are widely used in various applications, including nuclear radiation detection [4], biomedical applications, bio-sensing [5], in vivo imaging, energy conversion [6], and as photocatalysts [7]. However, comparatively slow luminescence decay and high self-absorption limit their use. In scintillators, self-absorption of emitted light reduces their overall efficiency, and re-emission of the absorbed light leads to longer decay times [8,9].

Unlike semiconductors, inorganic, solid-state scintillators with wide band gaps (~10 eV) generally exhibit low self-absorption. However, fast scintillators tend to have a low light yield—such as LuYSiO:Ce, which has a decay time of 40 ns and a light yield of 34 photons/keV. Conversely, scintillators with a high yield are relatively slow; for example, SrI_2_:Eu shows a 1 ms decay time with a yield of 120 ph./keV [10,11].

In contrast, a semiconductor in an InAs/GaAs quantum dot (QD) nanostructure proposed by [12,13] can be utilized as a scintillating medium, which is both fast and high yield. Using GaAs as a radiation-stopping material offers several advantages, including a direct band gap with a projected high photon yield (~240 ph./keV), reasonable radiation length, and high electron mobility, facilitating fast electron transport and capture in QDs (~2–5 ps). The self-assembled InAs QDs, which are used as artificial luminescence centers, exhibit radiative decays as short as 300 ps [14,15,16], making the InAs/GaAs QD system the fastest high-efficiency scintillator reported. The InAs QDs embedded in a GaAs matrix can be designed to minimize optical self-absorption, which is limited by the density of QDs (~10^15^ cm^−3^). Furthermore, absorption in GaAs is significantly reduced by the redshift (~250 nm) of the QD ground state luminescence with respect to the GaAs band edge. Additionally, the QD medium can be designed to provide a fast jitter of a few picoseconds, limited by the capture of electrons, and high QD luminescence efficiency at room temperature (>50%) [14].

The extraction of light from a semiconductor to an external photodetector (PD) is a well-known problem [17,18]. Because of the high refractive index *(n* = 3.5) of the GaAs’ matrix, only (2n)^−2^ = 2% of the photons emitted uniformly in space pass through a planar GaAs/air interface. The total internal reflection keeps the remaining photons inside the semiconductor. However, for III–V epitaxial films, the monolithic integration of a photodiode with a scintillator provides an alternative approach that enhances light extraction. As a result, the scintillator can then be turned into a waveguide (WG), and the coupling efficiency between the scintillator and the PD is no longer constrained by the high-index-contrast interface.

To fully utilize the unique speed and efficiency of the QD scintillation material, the integrated photodetector should have high absorption at the luminescent wavelength (as much as would be practical), a short signal propagation time, low capacitance, and low noise. The direct epitaxial growth of a p-i-n photodiode (PD) structure on top of the WG in a single epitaxial process and the etching away of portions of this PD structure both provide a convenient monolithic integration approach. While this technology does not yet yield detectors of a sufficient size and volume for efficient gamma-ray absorption and spectroscopy, the material properties and advanced semiconductors technology make it suitable for partitioning the detector into smaller pixels and leveraging its ultrafast characteristics [13]. In this work, we introduce design concepts for a monolithically integrated photodetector, specifically engineered to spectrally align with quantum dot luminescence. We also evaluate the detector’s performance using alpha and gamma radioactive sources, as well as its radiation hardness.

## 2. Materials and Methods

**Figure 1:**
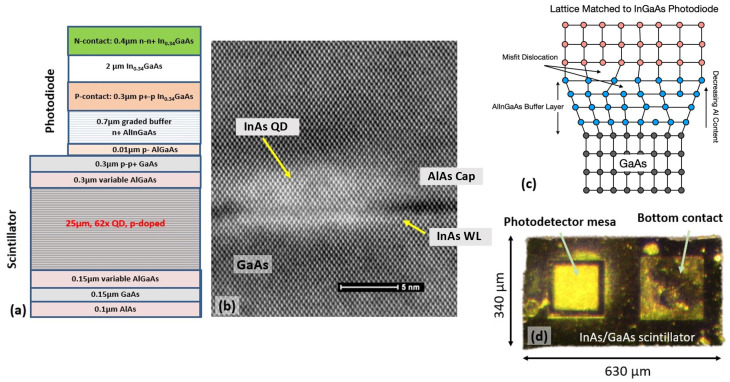
(**a**) Schematic structure of the InAs QDs/GaAs waveguiding scintillator and monolithically integrated InGaAs photodiode grown by MBE. (**b**) High-angle annular dark-field cross-sectional micrograph of an InAs QD along with an InAs wetting layer (WL) and 2ML thick AlAs capping layer. (**c**) Monolithic integration scheme for the GaAs matrix/waveguide and InGaAs photodetector with an AlInGaAs metamorphic buffer layer with variable Al content for gradual strain accommodation. (**d**) Top-down image of fabricated detector with one photodetector mesa and bottom contact.

The investigated structures were grown using Veeco (Tt.Paul, MN, USA) molecular beam epitaxy (MBE) on 3″ GaAs (001) substrates, forming 25 μm thick GaAs matrix layers embedded with sparse sheets of self-assembled InAs QDs. An integrated InGaAs p-i-n photodiode (PD) was grown on top of the scintillator structure (Figure 1a). The details of the epitaxial growth process are discussed elsewhere [15]. The growth began with a 100 nm thick AlAs sacrificial layer, which allowed the scintillation waveguide to be separated from the substrate using the epitaxial lift-off process [19]. To decrease photocarrier surface recombination, variable composition layers of Al_{0.3–0.1}_Ga_{0.7–0.9}_As were grown on both sides of the QD/WG as potential barriers.

The structure shown in Figure 1a consists of a 25 μm thick WG containing 62 sheets of InAs QDs grown at 515 °C and separated by 400 nm thick layers of GaAs matrix grown at 590 °C. The details of QD epitaxy with a reduced wetting layer were discussed previously [13]. A high-angle annular dark-field STEM micrograph of a QD is shown in Figure 1b, illustrating the MBE deposition sequence with capping of QDs by a two-monolayer-thick AlAs layer. This layer prevents evolution of the QDs on the growing surface, improving QD volume uniformity and, thus, luminescence efficiency [20], and eliminates long-wavelength absorption in the wetting layer states [21]. The InAs QDs are about 14 nm wide laterally and 4 nm tall, with a QD sheet density of 3–5 × 10^10^ cm^−2^. As a first pass optimization step, a 400 nm spacing was chosen, which was then raised to 800 nm. The QD bulk density and related self-absorption were reduced by increasing the spacing between the QD layers while maintaining a strong built-in electric field between them by engineering modulation p-type doping layers [15], which minimized electron drift time to just a few picoseconds [22]. The integrated PD is composed of In _0.35_Ga_0.65_As layers grown at 450 °C: a 400 nm n^+^-type contact layer followed by a 700 nm undoped absorber layer and a 300 nm p^+^-contact layer tuned to the QD emission wavelength. The PD structure is grown on a graded 700 nm metamorphic buffer layer (MBL) composed of p-type Al_(0.92–0.6)_In_(0.03–0.35)_Ga_0.05_As to accommodate a 2.5% misfit strain between GaAs and InGaAs with plastic deformation, as shown in Figure 1c. To minimize defect propagation, the structure was grown at a low temperature of 350 °C [23]. At room temperature, the band gap of the PD absorber was 0.95 eV, resulting in about a 60% absorption of the 1170 nm photons at a normal incidence.

Following the completion of the heterostructure growth of the InAs/GaAs scintillator detector in a single epitaxial process, as shown in Figure 1a, subsequent fabrication steps were applied to define the photodetector mesa. The process began with cleaning the sample by rinsing it with acetone and isopropanol to remove any residual surface contaminants. Next, the pattern for the top metal contact was defined using photolithography. Metal layers of Ti/Ni/Ag were then deposited onto the exposed surface using electron beam evaporation to form the n-type top contact. Excess metal was removed through a lift-off process by the MIF 300 solvent. Afterward, another photolithography step was performed to define the photodetector mesa. The mesa was formed in a two-step wet etching process. Firstly, the sample was etched in a piranha solution down to the MBL, followed by etching in buffered hydrofluoric acid that selectively stopped at the p-GaAs layer. The final photolithography step defined the bottom contact, followed by the deposition of a p-type metal layer (Pd/Ti/Pd/Ag). After the fabrication process, the devices were separated from the parent wafer using an epitaxial lift-off process. The lifted-off devices, approximately 25 µm thick, were then cleaved into individual units for further characterization and measurement. The final device is illustrated in Figure 1c. The detector response is read through the top contact while the bottom contact is grounded.

Transmission electron microscopy (TEM) was used to examine the microstructure of the material, photoluminescence was employed to evaluate the optical properties of the QD waveguides, and ^241^Am and ^57^Co radioactive sources were used to study the detector response to the 5.5 MeV α-particles and the 122 keV photons. The detectors were mounted onto a custom-designed printed circuit board (PCB) utilizing flip-chip bonding. This method reduces the parasitic capacitance and inductance, while also simplifying detector handling. The PCB was then connected to a readout board featuring a Cremat charge-sensitive amplifier and a 1 μs shaping amplifier. The output was read by a digital oscilloscope. The data were collected with a trigger threshold of approximately 3 times the noise standard deviation (3σ_noise_). The radiation hardness of the QDs was evaluated using a 1 MeV proton beam with implanted doses of up to 10^14^ protons/cm^2^.

## 3. Results and Discussion

### 3.1. Design of an Integrated Photodetector

**Figure 2:**
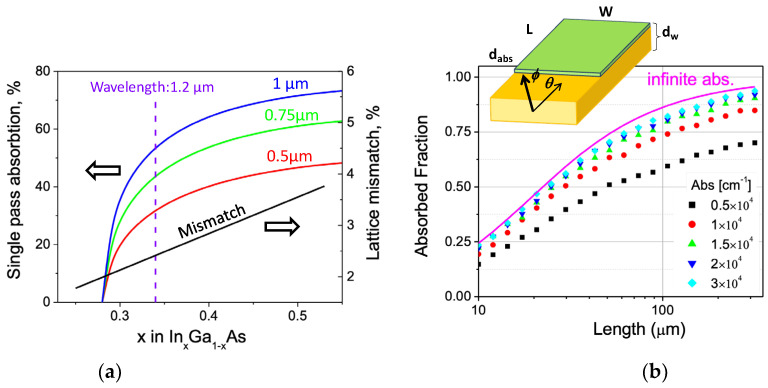
(**a**) Single pass normal absorption of 1.2 μm light in In_x_Ga_1-x_As layers of various thicknesses and the associated misfit strain on a GaAs substrate. The dashed line indicates the target absorption of the integrated photodetector. The necessary InGaAs absorber thickness is well above the critical thickness for dislocation nucleation at x = 0.34; therefore, a metamorphic buffer is placed between the GaAs WG and the PD. (**b**) 3D Monte Carlo simulations of an integrated PD coupling with a 25 μm thick multimode waveguide as a function of PD length. Solid line shows the asymptotic behavior for an infinite PD absorption.

Due to the high refractive index of GaAs, the most straightforward solution for efficient PD coupling was a monolithic integration with a waveguide. Efficient absorption of QD luminescence at λ = 1.2 μm requires a PD material with a band gap below 1 eV. Figure 2a estimates a single-pass absorption at a normal incidence in In_x_Ga_1-x_As layers with varying compositions. To achieve practical PD efficiency, the indium content in the alloy must exceed x > 0.35. However, this alloy introduces a lattice mismatch of >2.5% with GaAs, and the detector thickness and composition exceed the critical limits for dislocation nucleation. Therefore, a design incorporating a relatively thick MBL is required.

The PD design was further optimized using Monte Carlo simulations to model the absorption of the waveguided light in a 1 μm thick PD. The optical coupling was modeled using a ray optics approximation, which is accurate for thick multimode waveguides, and neglecting losses in the MBL. The latter assumption was confirmed by the recent results on interference in the GaAs/MBL structure, confirming that the losses were mostly related to the interface reflection that introduced a reflection of about 0.5% at a normal incidence [24], which makes this structure good enough for optical coupling with the PD.

The results shown in Figure 2b indicate that to absorb at least half of the QD luminescence, the In_0.35_Ga_0.65_As absorber (absorption coefficient ~(0.8–1.2) × 10^4^ cm^−1^) should be longer than 30 μm along the WG.

**Figure 3:**
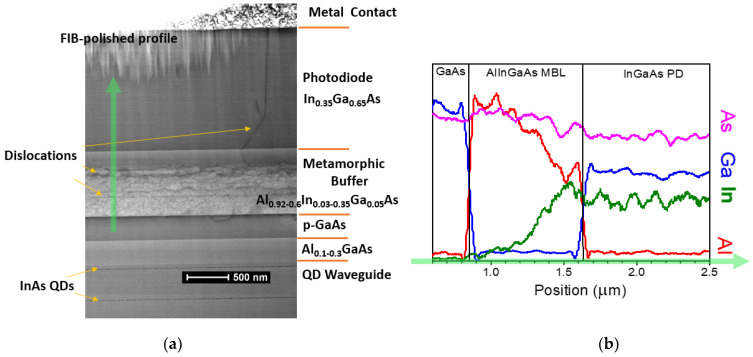
(**a**) Bright-field (110) STEM cross-sectional micrograph of the top section of the detector. The 0.8 μm AlInGaAs metamorphic buffer layer, with varying composition to accommodate the lattice mismatch between the GaAs waveguide and the In_0.35_Ga_0.65_As photodiode (PD) layers, exhibits a high dislocation density. Dislocations are effectively captured in the graded buffer layer, limiting treading dislocation density in the PD. (**b**) Characteristic X-ray intensity profiles (energy-dispersive spectroscopy) to illustrate composition changes along the scan are shown in (**a**) by a thick arrow.

**Figure 4:**
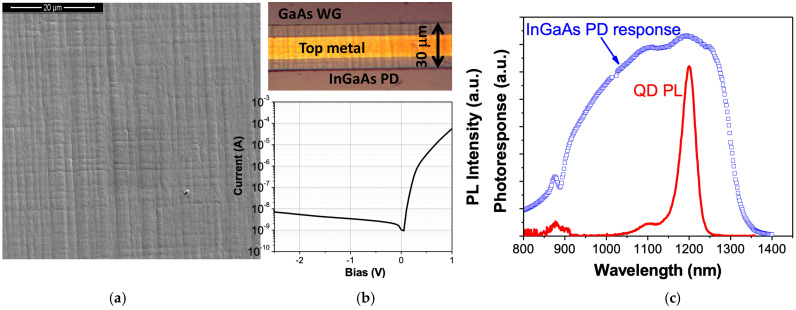
(**a**) SEM image of the PD surface showing the crosshatch morphology of the metamorphic InGaAs/GaAs layer. (**b**) The InGaAs photodiode processed on the GaAs WG (top); IV characteristic of a fabricated InGaAs diode on a metamorphic buffer on top of the GaAs scintillation waveguide (bottom) measured at room temperature. (**c**) Room temperature spectra of QD photoluminescence, along with the InGaAs photodetector photosensitivity curve.

The most challenging part of the integrated PD is its relatively thin MBL, which must not introduce significant optical losses. We used a 0.8 μm thick metamorphic buffer, which is shown in the STEM micrograph in Figure 3a. The buffer consists of a quaternary AlInGaAs compound with a fixed 5% Ga content and varying In/Al ratio to gradually accommodate the lattice mismatch between GaAs and In_0.35_Ga_0.65_As [23]. The variation in composition is illustrated by a characteristic X-ray profile (Figure 3b). The MBL has high dislocation density; however, these dislocations are effectively trapped within the graded layer. Cross-sectional micrographs with a field of view of a few micrometers typically reveal one or two threading dislocations (Figure 3a), providing a rough estimate of the dislocation density, (1–3) × 10^7^ cm^−2^. Some dislocations from the buffer layer extend into the waveguide as a result of high misfit stress accumulated during growth. A characteristic feature of the metamorphic nature of the absorber growth is a crosshatch surface morphology in the growing film [25], as shown in Figure 4a. This pattern results from the strain associated with misfit dislocations in the buffer. The surface morphology remains evident even on the PD contact metal layer, as illustrated at the top of Figure 4b. Threading dislocations in the absorber—and, possibly, the growth morphology—affect the parameters of the PD. Figure 4b shows the room temperature current–voltage characteristics of the PD with a relatively high reverse current in the 0.1 mA/cm^2^ range. Although this value is 2–3 times lower than the best reported values for metamorphic In_0.53_Ga_0.47_As/GaAs PDs [26,27], our devices had a wider band gap and were expected to have a dark current at least an order of magnitude lower, with potential for further reduction through optimization of the epitaxial growth process of the MBL. Nevertheless, the achieved values are quite reasonable, considering the uncommonly high overall thickness of the MBE-grown strained structure (over 25 mm) and the relatively thin MBL, which was selected to enhance coupling with the waveguide. This PD design has previously demonstrated time responses below 50 ps [15]. In this study, we used the PD with a zero external bias to reduce dark current noise.

Optoelectronic behavior of the integrated PD is demonstrated by Figure 4c, where the maximum of the PD spectral response coincides with the room temperature QD PL band. It can be concluded that the PD design looks appropriate for the scintillation detector’s assembly and testing.

### 3.2. Photoluminescence Collection Efficiency with an Integrated Photodetector

**Figure 5:**
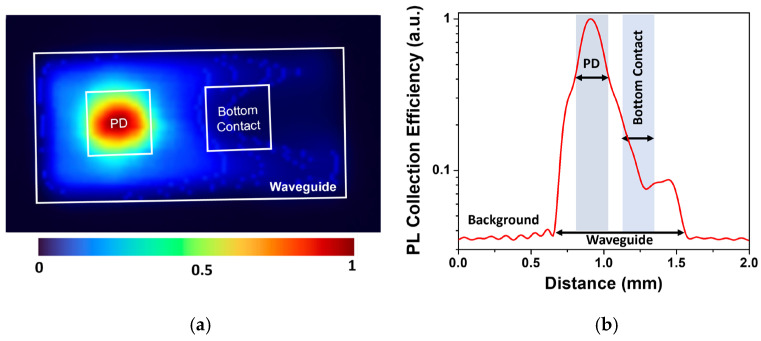
(**a**) PL collection efficiency map for a 920 μm × 390 μm sample with a 300 × 300 μm^2^ PD mesa and bottom 280 × 280 μm^2^ PD contact. The white boundary represents the edge of the waveguide (**b**) 1D PL scan passing though the PD and bottom contact along the center line of the WG (color online).

The waveguiding properties of the device were studied using 2D scanning photoluminescence (PL) measurements. A CW 10 mW 520 nm ThorLabs (Newton, NJ, USA) multichannel laser source was used for the excitation. The QD waveguide was placed on a glass slab to allow the laser beam to pass through and excite the scintillator. The integrated PD collected the photocurrent while the laser beam was scanned across the opposite surface of the waveguide. Thus the beam provided surface excitation limited to a few tens of nanometers in the GaAs, preventing any direct PD exposure. The resulting 2D PL map of the device is shown in Figure 5a. The maximum luminescence signal was observed when the laser beam was directly beneath and around the PD mesa. Near the perimeter of the PD mesa, the charge collection efficiency dropped roughly by half, as shown in Figure 5b. This initial drop in efficiency is attributed to the equal probability of PL being emitted towards and away from the PD. In the latter case, the scintillation light has mostly vanished due to absorption and scattering in the WG. It should be noted that, due to efficient total internal reflection, light typically travels about twice the lateral distance of the waveguide, bouncing roughly nine times for 300 μm of lateral travel. The PL collection efficiency drops at an increased excitation distance from the PD, mostly due to scattering and self-absorption. The absorption spectrum of similar QDs was previously assessed by analyzing the evolution of the PL spectrum with varying waveguiding lengths [15]. Self-absorption was found to be around 1 cm^−1^ over the wavelength range of ~80 nm, coinciding with the ground state QD luminescence, and increased sharply at shorter wavelengths, corresponding to the excited state QD emission. Notably, the typically strong absorption in the wetting layer is nearly eliminated due to capping the dots with an AlAs layer [21]. Summarizing the present results, we conclude that light scattering is the primary contributor to optical losses as a function of waveguiding length in this system.

A significant loss in the PL collection efficiency was observed in the region of the bottom contact of the PD (semiconductor/metal interface), where the collection efficiency fell below 10%. This issue could be mitigated by reducing the contact area. The light collection efficiency averaged over the detector area was estimated to be around 10%. Due to the device geometry, the response is expected to exhibit a multi-modal distribution: a high-collection efficiency peak beneath the PD area, the second lower-efficiency peak corresponding to the WG area around the PD, and the remaining areas of the WG screened by the bottom contact.

### 3.3. Detector Response to α-Particles and Photons

**Figure 6:**
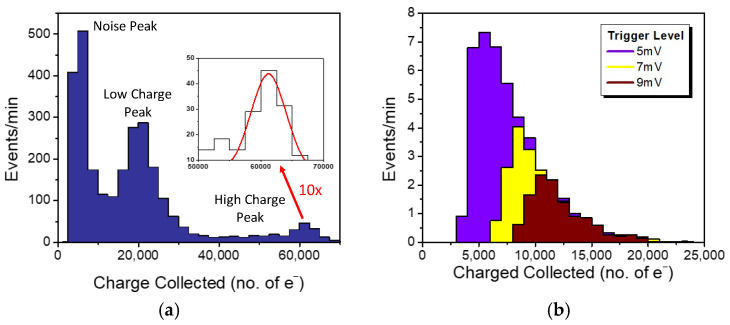
Histograms of charges collected by the waveguiding scintillation detector with integrated 300 × 300 μm^2^ PDs from (**a**) ^241^Am α-particles and (**b**) ^57^Co photons taken at three different trigger levels.

The detector response to different types of radiation is evaluated with 5.5 MeV α-particles from ^241^Am (1 μCi) and 122 keV photons from ^57^Co (40 μCi) sources. The detector was read out by a charge-sensitive amplifier followed by a shaping amplifier with a 10× gain. The gain of the readout board was evaluated using a pulse generator with a value of 10^13^ VC^−1^ and a sensitivity of 625 electrons/mV. The oscilloscope trigger threshold was set to ensure no signal was detected without the radioactive source. Figure 6a shows the distribution of collected charges from α-particles. As mentioned in Section 3.2, the detector geometry was expected to produce a multi-modal charge distribution. The high-charge peak, with a mean of 62,000 electrons, corresponds to alpha-particles absorbed in the WG beneath the PD mesa. The source was positioned 5 mm away from the detector, and accounting for energy losses in the air, the energy of the alpha-particles reaching the detector was estimated to be 4.8 MeV. As a result, an average signal of about 13,000 electrons/MeV was collected. The energy resolution of 12% FWHM was likely determined by the detector’s geometry.

Figure 6b shows the charge distribution collected with 122 keV gamma photons. The detector response was measured at three trigger levels, with the lowest level of 5 mV corresponding to 3100 electrons, allowing for the capture of some noise pulses. There were no pulses detected without the ^57^Co source at the two higher trigger levels. Unlike the α-particle response, the photon charge distributions were significantly shaped by the readout threshold. The tail of the charge distributions remained consistent across different trigger thresholds, indicating that only the tail was detectable. Moreover, most responses to 122 keV photons were obscured by the readout noise level. In the tail region, a charge collection greater than 40,000 electrons/MeV was observed. The collected charge was significantly higher for photons than for alpha-particles. Many inorganic and organic scintillator materials exhibit Birks’s quenching, explaining the non-linear response between photons and charged particles due to saturation of the scintillation yield at high deposition energy densities [28].

**Figure 7:**
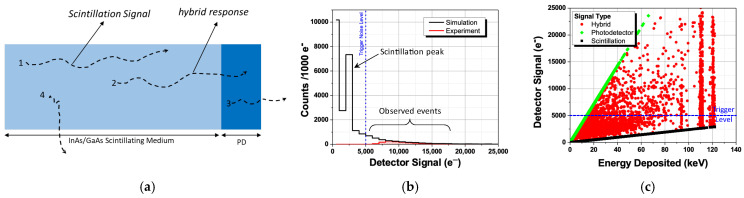
(**a**) Possible mechanisms of gamma-signal formation in the InAs QD scintillation detector with an integrated PD. Dashed lines indicate the paths of primary photoelectrons. (**b**) The Geant4 simulated response of a 25 μm GaAs with a 3 μm integrated InGaAs photodetector to 122 keV gamma photons with a 10% overall scintillation detector efficiency. (**c**) The detector signal corresponding to energy deposited by 122 keV gamma photons via mechanisms (1) to (3). Note that direct PD ionization (mechanism 3—green dots) is responsible for about 15% of the experimentally observed events in Figure 6b, with 85% of the events produced by the hybrid response.

In our detector, the discrepancy between the alpha-particle and photon responses arose from different energy deposition patterns. For 4.8 MeV alpha-particles, the energy was entirely deposited within the 25 μm thick InAs/GaAs scintillating medium, and the observed detector response was attributed solely to scintillation. However, for 122 keV photons, their interactions with the materials were much weaker, with only about 0.5% of the photons absorbed by the thin scintillator. The dominant interaction mechanism was the photoelectric effect, with a 61% probability [29]. The projected range of the resultant photoelectrons was approximately 53 μm, meaning that they can either travel from the scintillator to the photodetector or escape the scintillator, leading to partial energy deposition. Thus, the energy deposition can be divided into four categories, as shown in Figure 7a: (1) energy is fully deposited in scintillating medium, (2) the deposited energy is shared between the scintillating medium and direct ionization of the PD, (3) energy is deposited only in the PD (likely partially due to the small thickness) via direct ionization, and (4) partial energy deposited in the scintillating medium. The hybrid response occurs when the photoelectron’s energy is shared between the scintillating medium and the integrated PD, and can be estimated as

$${Detector}_{Signal}=QD_{Signal}+PD_{Signal}$$

where

$$QD_{Signal}\;=\;\frac{E_{Deposited\;in\;InAs/GaAs}}{4.2\;\;\mathrm{eV}}\;\times \;overall\;QDSD\;efficiency,$$

and

$$PD_{Signal}=\frac{E_{Deposited\;in\;PD}}{2.7\;\mathrm{eV}}\times PD\;efficiency$$

where $2.7\;eV=2.8\times E_{g,PD}$ is the average energy spent to generate an electron–hole pair in InGaAs with the band gap *E_g_*_,*PD*_ = 0.95 eV.

A Geant4 [30] simulation was performed to model the detector response based on energy deposited in both the scintillating medium and the PD for each photon interaction. The simulation only considered interactions within the scintillating medium covered by the 300 μm × 300 μm PD. The simulated detector’s response to the 122 keV gamma photons is shown in Figure 7b. The overall scintillation detector efficiency, determined to be 10% based on alpha-particle measurements, was applied in the analysis, and the detector response was scaled by aligning the slope of experimental results above 8000 electrons. The direct ionization efficiency of the PD was assumed to be 100%.

The simulated detector response exhibited a distinct peak at 2500 electrons, corresponding to the 122 keV photopeak. This peak arose from events in which the photoelectron’s energy was fully contained within the 25 μm scintillating medium (process 1), indicating that it was predominantly driven by the scintillation signal. The tail following this peak results from events where the deposited energy was shared between the scintillating medium and the PD, resulting in a hybrid signal (process 2). These simulation results help clarify the experimental findings showing that the scintillation signal was partially masked by the noise level of 5000 electrons. Additionally, the fact that the response tail remained constant, above 7500 electrons, across different trigger levels suggests that this portion of the histogram is dominated by the hybrid response of the detector (Figure 7c). It should be noted that the hybrid signal only surpassed the scintillation signal due to the detector’s relatively low efficiency—10% in the current structure. With improved light collection efficiency, the scintillation signal should surpass the hybrid response, highlighting the significant potential of the InAs QD scintillating medium, even with the limitations posed by the detector’s small volume.

### 3.4. Radiation Hardness Measurements

**Figure 8:**
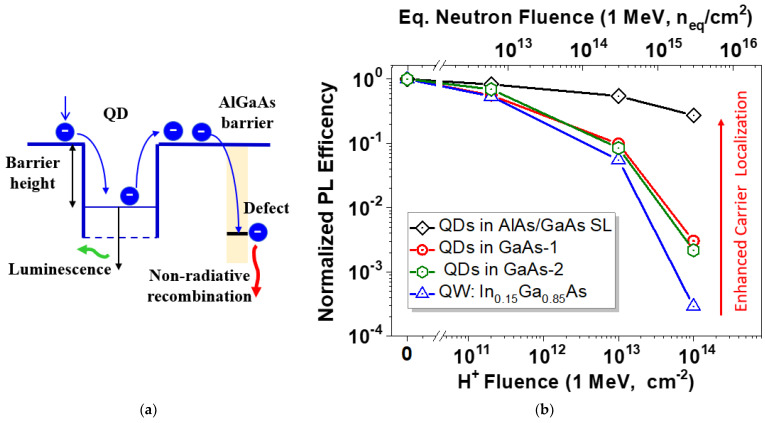
(**a**) Schematic band diagram of the QD structure with electron capture/escape processes in a p-type material. (**b**) Effect of 1 MeV proton irradiation on photoluminescence efficiency of different QD structures and a quantum well structure for comparison. The top axis shows the calculated NIEL-equivalent neutron fluence.

Radiation defects in a semiconductor scintillator primarily appear as carrier traps with energy levels in the band gap, acting as non-radiative recombination centers. Once formed, they compete with the QDs for the carriers, increasing the probability of non-radiative recombination and resulting in a loss of scintillation efficiency. Our previous studies demonstrated that at or above room temperature, PL quenching in QD structures from radiation defects is primarily caused by carrier escape from the QDs to the barrier (Figure 8a), which serves as a material with a low damage constant [31]. In fact, the relatively high barrier for carrier escape (up to 450 meV for an AlGaAs barrier) and the low non-radiative recombination rate in QDs both contribute to their exceptional tolerance to defects and ability to maintain high PL efficiency at both room and elevated temperatures.

The InAs QDs can be hosted within matrix materials with different barrier heights. To assess the radiation hardness of the QDs, four types of samples were prepared: two samples with slightly different InAs QDs embedded in a GaAs matrix, which were similar to the scintillators discussed above; one sample with QDs embedded in a wider band gap AlAs/GaAs short-period superlattice corresponding to the Al_0.25_Ga_0.75_As alloy; and a final sample that uses the In_0.15_Ga_0.85_As/GaAs quantum well (QW) structure for reference. The samples were cleaved from 3-inch wafers and irradiated with a 1 MeV proton (H^+^) beam up to a dose of 10^14^ protons/cm^2^. Figure 8b shows the relationship between the integrated PL and the implanted dose. The InGaAs QW structure experienced the greatest PL quenching, followed by the structures with a GaAs matrix. In contrast, the QDs embedded into the wide band gap AlGaAs tolerated doses 100 times higher than those in the GaAs matrix. To extrapolate the proton fluences to the standardized radiation environment, we used the established value for cross-sections for non-ionizing energy losses (NIELs) in GaAs, which is 60 keVˑcm^2^/g for 1 MeV protons [32,33]. Accordingly, the 1 MeV neutron equivalent fluence for the displacement damage was 29.5 times higher than for protons. Therefore, the InAs/AlGaAs QD scintillator is expected to withstand about (1–3) × 10^15^ n_eq_/cm^2^, comparable to or slightly better than the radiation hardness of inorganic solid-state scintillators [34] and Si strip detectors [35].

## 4. Conclusions

A novel scintillation material featuring artificial luminescence centers, InAs quantum dots, was developed alongside a monolithically integrated p-i-n photodiode. The design methodology for integrating a photodetector with a waveguiding scintillator was presented. The PL collection efficiency was evaluated using 2D scanning PL measurements and revealed the impact of device geometry, with the highest PL collection observed from the region beneath the PD. The device’s response was measured with both α-particles and photons. For 4.8 MeV α-particles, the charge collection was measured at 13,000 electrons/MeV with an energy resolution of 12%. For 122 keV photons, the collected charge exceeded 40,000 electrons/MeV. The difference in detector signals for photons and alpha-particles is attributed to their energy deposition patterns with or without direct ionization of the PD, respectively. The radiation hardness of QD-based scintillators was evaluated using a 1 MeV proton beam up to a dose of 10^14^ protons/cm^2^. The QDs embedded in the AlAs/GaAs short-period superlattice matrix corresponding to the Al_0.35_Ga_0.65_As alloy showed a 100× improvement in the radiation hardness over the QDs in the GaAs matrix. This improvement was due to the enhanced electron localization in a wider band gap barrier.

## Data Availability

Data are available upon request.

## References

[1] Lecoq P. (2016). Development of New Scintillators for Medical Applications. Nucl. Instrum. Methods Phys. Res. Sect. A Accel. Spectrometers Detect. Assoc. Equip..

[2] Wang Z., Guardincerri E., Rathman D.D., Azzouz M.E., Barnes C.W., Berger R., Bond E.M., Craig D.M., Holtkamp D., Kapustinsky J.S. (2013). Gigahertz (GHz) Hard X-Ray Imaging Using Fast Scintillators. Proc. SPIE.

[3] Glodo J., Wang Y., Shawgo R., Brecher C., Hawrami R.H., Tower J., Shah K.S. (2017). New Developments in Scintillators for Security Applications. Phys. Procedia.

[4] Derenzo S.E., Bourret-Courshesne E., Bizarri G., Canning A. (2016). Bright and Ultra-Fast Scintillation from a Semiconductor?. Nucl. Instrum. Methods Phys. Res. Sect. A Accel. Spectrometers Detect. Assoc. Equip..

[5] Zhao L., Hu L., Fang X. (2012). Growth and Device Application of CdSe Nanostructures. Adv. Funct. Mater..

[6] Zhang Q., Li H., Ma Y., Zhai T. (2016). ZnSe Nanostructures: Synthesis, Properties and Applications. Prog. Mater. Sci..

[7] Lee G.-J., Wu J.J. (2017). Recent Developments in ZnS Photocatalysts from Synthesis to Photocatalytic Applications—A Review. Powder Technol..

[8] Popescu D.P., Eliseev P.G., Stintz A., Malloy K.J. (2003). Carrier Migration in Structures with InAs Quantum Dots. J. Appl. Phys..

[9] Luryi S., Subashiev A., Luryi S., Xu J., Zaslavsky A. (2010). Semiconductor Scintillator for Three-Dimensional Array of Radiation Detectors. Future Trends in Microelectronics.

[10] Knoll G.F. (2010). Radiation Detection and Measurement.

[11] https://scintillator.lbl.gov/inorganic-scintillator-library/.

[12] Luryi S. (2008). Impregnated Semiconductor Scintillator. Int. J. High Speed Electron. Syst..

[13] Oktyabrsky S., Yakimov M., Tokranov V., Murat P. (2016). Integrated Semiconductor Quantum Dot Scintillation Detector: Ultimate Limit for Speed and Light Yield. IEEE Trans. Nucl. Sci..

[14] Dropiewski K., Minns A., Yakimov M., Tokranov V., Murat P., Oktyabrsky S. (2020). Ultrafast Waveguiding Quantum Dot Scintillation Detector. Nucl. Instrum. Methods Phys. Res. Sect. A Accel. Spectrometers Detect. Assoc. Equip..

[15] Dropiewski K., Minns A., Yakimov M., Tokranov V., Murat P., Oktyabrsky S. (2020). Optical Properties of InAs Quantum Dots/GaAs Waveguides for Ultra-Fast Scintillators. J. Lumin..

[16] Minns A., Dropiewski K., Yakimov M., Tokranov V., Hedges M., Murat P., Oktyabrsky S. (2021). Parameters of Fast and High-Yield InAs/GaAs Quantum Dot Semiconductor Scintillator. MRS Adv..

[17] Joyce W.B., Bachrach R.Z., Dixon R.W., Sealer D.A. (1974). Geometrical Properties of Random Particles and the Extraction of Photons from Electroluminescent Diodes. J. Appl. Phys..

[18] Zhmakin A.I. (2011). Enhancement of Light Extraction from Light Emitting Diodes. Phys. Rep..

[19] Yablonovitch E., Gmitter T., Harbison J.P., Bhat R. (1987). Extreme Selectivity in the Lift-off of Epitaxial GaAs Films. Appl. Phys. Lett..

[20] Tokranov V.E., Yakimov M., Katsnelson A., Lamberti M., Oktyabrsky S. (2003). Shape Engineered InAs Quantum Dots with Stabilized Electronic Properties. Proc. SPIE.

[21] Varghese A., Yakimov M., Tokranov V., Mitin V., Sablon K., Sergeev A., Oktyabrsky S. (2016). Complete Voltage Recovery in Quantum Dot Solar Cells Due to Suppression of Electron Capture. Nanoscale.

[22] Kasamatsu N., Kada T., Hasegawa A., Harada Y., Kita T. (2014). Effect of Internal Electric Field on InAs/GaAs Quantum Dot Solar Cells. J. Appl. Phys..

[23] Lubyshev D., Liu W.K., Stewart T.R., Cornfeld A.B., Fang X.M., Xu X., Specht P., Kisielowski C., Naidenkova M., Goorsky M.S. (2001). Strain Relaxation and Dislocation Filtering in Metamorphic High Electron Mobility Transistor Structures Grown on GaAs Substrates. J. Vac. Sci. Technol. B Microelectron. Nanometer Struct. Process. Meas. Phenom..

[24] Minns A., Mahajan T., Tokranov V., Yakimov M., Hedges M., Murat P., Oktyabrsky S. (2024). Device Response Principles and the Impact on Energy Resolution of Epitaxial Quantum Dot Scintillators with Monolithic Photodetector Integration. Sci. Rep..

[25] Romanato F., Napolitani E., Carnera A., Drigo A.V., Lazzarini L., Salviati G., Ferrari C., Bosacchi A., Franchi S. (1999). Strain Relaxation in Graded Composition InxGa_1−x_As/GaAs Buffer Layers. J. Appl. Phys..

[26] Jang J.-H., Cueva G., Hoke W.E., Lemonias P.J., Fay P., Adesida I. (2002). Metamorphic Graded Bandgap InGaAs-InGaAlAs-InAlAs Double Heterojunction p-i-I-n Photodiodes. J. Light. Technol..

[27] Hoke W.E., Lemonias P.J., Kennedy T.D., Torabi A., Tong E.K., Bourque R.J., Jang J.-H., Cueva G., Dumka D.C., Adesida I. (2001). Metamorphic Heterojunction Bipolar Transistors and P–I–N Photodiodes on GaAs Substrates Prepared by Molecular Beam Epitaxy. J. Vac. Sci. Technol. B Microelectron. Nanometer Struct. Process. Meas. Phenom..

[28] Birks J.B. (1951). Scintillations from Organic Crystals: Specific Fluorescence and Relative Response to Different Radiations. Proc. Phys. Soc. A.

[29] Berger M., Hubbell J., Seltzer S., Coursey J., Zucker D. (1999). XCOM: Photon Cross Section Database (Version 1.2). XCOM: Photon Cross Section Database (Version 1.2). http://physics.nist.gov/xcom.

[30] Agostinelli S., Allison J., Amako K., Apostolakis J., Araujo H., Arce P., Asai M., Axen D., Banerjee S., Barrand G. (2003). G_eant_4—A Simulation Toolkit. Nucl. Instrum. Methods Phys. Res. Sect. A Accel. Spectrometers Detect. Assoc. Equip..

[31] Oktyabrsky S., Lamberti M., Tokranov V., Agnello G., Yakimov M. (2005). Room-Temperature Defect Tolerance of Band-Engineered InAs Quantum Dot Heterostructures. J. Appl. Phys..

[32] Summers G.P., Burke E.A., Xapsos M.A., Dale C.J., Marshall P.W., Petersen E.L. (1988). Displacement Damage in GaAs Structures. IEEE Trans. Nucl. Sci..

[33] Akkerman A., Barak J., Chadwick M.B., Levinson J., Murat M., Lifshitz Y. (2001). Updated NIEL Calculations for Estimating the Damage Induced by Particles and γ-Rays in Si and GaAs. Radiat. Phys. Chem..

[34] Yang F., Zhang L., Zhu R.-Y., Kapustinsky J., Nelson R., Wang Z. (2016). Proton Induced Radiation Damage in Fast Crystal Scintillators. Nucl. Instrum. Methods Phys. Res. Sect. A Accel. Spectrometers Detect. Assoc. Equip..

[35] Hara K., Allport P.P., Baca M., Broughton J., Chisholm A., Nikolopoulos K., Pyatt S., Thomas J.P., Wilson J.A., Kierstead J. (2016). Charge Collection and Field Profile Studies of Heavily Irradiated Strip Sensors for the ATLAS Inner Tracker Upgrade. Nucl. Instrum. Methods Phys. Res. Sect. A Accel. Spectrometers Detect. Assoc. Equip..

